# Comparative Chloroplast Genomics of *Sophora* Species: Evolution and Phylogenetic Relationships in the Early-Diverging Legume Subfamily Papilionoideae (Fabaceae)

**DOI:** 10.3389/fpls.2021.778933

**Published:** 2021-12-16

**Authors:** Min Liao, Xin-Fen Gao, Jun-Yi Zhang, Heng-Ning Deng, Bo Xu

**Affiliations:** ^1^CAS Key Laboratory of Mountain Ecological Restoration and Bioresource Utilization and Ecological Restoration and Biodiversity Conservation Key Laboratory of Sichuan Province, Chengdu Institute of Biology, Chinese Academy of Sciences, Chengdu, China; ^2^College of Life Sciences, University of Chinese Academy of Sciences, Beijing, China

**Keywords:** gene loss, IR expansion, molecular markers, relocation, *Sophora*, plastome

## Abstract

The taxonomy and evolutionary history of *Sophora* L., a genus with high economic and medicinal value, remain uncertain due to the absence of genetic resource (especially in China) and low polymorphism of molecular markers. Our aim was to elucidate the molecular evolution and phylogenetic relationships in chloroplast genomes of *Sophora* species in the early-diverging legume subfamily Papilionoideae (Fabaceae). We reported nine *Sophora* chloroplast genome from China using Illumina sequencing. We performed a series of analyses with previously published genomes of *Sophora* species to investigate their genomic characteristics, identified simple sequence repeats, large repeat sequences, tandem repeats, and highly polymorphic loci. The genomes were 152,953–158,087 bp in length, and contained 111–113 unique genes, including 76–78 protein coding, 31 tRNA, and 4 rRNA. The expansion of inverted repeat boundary of *Sophora* resulted in *rps12* entering into the LSC region and loss of *trnT*-*CGU* gene in some species. Also, we found an approximately 23 kb inversion between *trnC-GCA* and *trnF-GAA* within the genus. In addition, we identified seven highly polymorphic loci (pi (π) > 0.035) suitable for inferring the phylogeny of *Sophora* species. Among these, three regions also co-occurred with large repeat sequences and support use of repeats as a proxy for the identification of polymorphic loci. Based on whole chloroplast genome and protein-coding sequences data-set, a well-supported phylogenetic tree of *Sophora* and related taxa showed that this genus is monophyletic, but sect. *Disamaea* and sect. *Sophora*, are incongruent with traditional taxonomic classifications based on fruit morphology. Our finding provides significant genetic resources to support further investigation into the phylogenetic relationship and evolution of the genus *Sophora*.

## Introduction

The plastid genome (plastome) of photosynthetic flowering plants is generally extremely conserved in terms of structural organization, gene content (115–160 genes), gene arrangement, and GC content (34–40%; Palmer, 1985; Mower and Vickrey, 2018). The plastome is generally a quadripartite structure comprised of a large single-copy region (LSC), a small single-copy region (SSC), and two inverted repeat (IR) regions (Abdullah et al., 2019; Abdullah, Henriquez et al., 2020a; Guo et al., 2021). Plastome sequences have been widely used in studies of phylogeny, evolution, and population genetics of flowering plants (Tonti-Filippini et al., 2017). With the development of high-throughput sequencing technology, the number of available complete chloroplast genomes has increased dramatically (Sinn et al., 2018; Lee et al., 2020; Zhang et al., 2020b; Guo et al., 2021). Several mutational events occur in plastomes, including indels, inversions, substitutions, contractions, and expansions of the inverted repeats. These mutations affect the gene content of plastomes through gene duplication, gene loss, and pseudogenization (Guisinger et al., 2011; Vieira et al., 2014; Abdullah, Mehmood, et al., 2020a; Alqahtani and Jansen, 2021). Growing evidence have been found about plastid rearrangements in photosynthetic angiosperms. For example, rearrangements have been found in the Asteraceae (Kim et al., 2005; Sablok et al., 2019), Bignoniaceae (Fonseca and Lohmann, 2017), Campanulaceae (Haberle et al., 2008; Uribe–Convers et al., 2017), Fabaceae (Lavin et al., 1990; Cai et al., 2008; Schwarz et al., 2015; Keller et al., 2017; Wang et al., 2018; Jin et al., 2019; Oyebanji et al., 2020), Geraniaceae (Wicke et al., 2011; Röschenbleck et al., 2017), Oleaceae (Lee et al., 2007), Plantaginaceae (Zhu et al., 2016; Asaf et al., 2020), and Poaceae (Burke et al., 2016; Liu et al., 2020).

Plastomes in the legume family range from 123–180 kb (LSC: 71,912–112,248 kb; SSC: 13,632–59,438 kb; IR: 23,489–41,968 kb) in length, with considerable variation due to expansion or contraction of the IR region, or loss of the inverted copy (Wang et al., 2018; Zhang et al., 2020a). Smaller plastomes that have lost the IR are known as IRLC (inverted repeat-lacking clade) in this family (Wojciechowski et al., 2000). The larger plastid genomes are usually characterized by inverted repeat expansion (Dugas et al., 2015; Wang et al., 2017). For example, the tribe Ingeae has IRs expansion of around 13 kb toward the SSC. Growing evidence have been found about inversions within this family. For example, two papilionoid tribes, Swartzieae and Sophoreae, were found to have a 50 kb inversion in the LSC region (Doyle et al., 1996). Also, a 23, 24, or 36 kb have been described in various genera of the genistoid clade (Martin et al., 2014; Choi and Choi, 2017; Feng et al., 2017; Keller et al., 2017) and ~ 23 kb inversion between *trnC*-*GCA* and *trnF*-*GAA* was first reported in *Sophora alopecuroides* L. (Zha et al., 2020). The genes *accD*, *ndhD*, *psaI*, *rpl23*, *rpl32*, *rpl33*, *rps16, rps18, rps19*, and *ycf4*, have been functionally lost in the Papilionoideae and numerous other legume lineages (Schwarz et al., 2015; Keller et al., 2017; Oyebanji et al., 2020; Zha et al., 2020). In addition, one or two introns have been lost from *clpP*, *rpl2*, *rps12*, and *rps16* in many legume lineages (Doyle et al., 1995; Jansen et al., 2008; Dugas et al., 2015; Wang et al., 2017).

*Sophora* L. is a medicinally important genus of the subfamily Papilionoideae (Fabaceae), containing appropriately 50–70 species, which are mainly distributed in tropical and temperate regions (Pennington et al., 2005; Mattapha et al., 2018). Study of active components from plants of this genus have shown potential of antitumor, anti-inflammatory, anti-arrhythmia, antibacterial, antitoxin, and immune regulation properties (Zhang et al., 2014; Chen et al., 2020). Meanwhile, some species play vital roles in biological nitrogen fixation, and soil conservation and restoration (Iinuma et al., 1995; Liang et al., 2012). Classical taxonomic studies of *Sophora* have depended on its leaf morphology, fruit morphology, and seed color (Tsoong and Ma, 1981; Ma, 1990). However, the morphological features are greatly affected by the environmental conditions. The complexity of these morphological characters has made it challenging to understand *Sophora* taxonomy and evolution (Hurr et al., 1999; Mitchell and Heenan, 2002; Heenan et al., 2004; Shepherd and Heenan, 2017, 2021; Duan et al., 2019). Recently, researchers focused on species located in New Zealand, where chloroplast and nuclear evidence do not conflict, but low bootstrapping support was observed for various nodes. Shepherd and Heenan (2021) attributed it to hybridization and introgression of species based on genome-wide SNP data from the region. Duan et al., (2019) stated that *Sophora* is not monophyletic based on ITS and plastid markers (*matK*, *psbA*-*trnH* and *trnL*-*F*). Asia, as one of the centers of distribution, phylogenetic relationships of *Sophora* species are poorly known. Therefore, a robust backbone phylogeny of the genus *Sophora* has not been constructed due to the lack of sufficient genetic resources. The resolution of such relationships will be of great guidance for taxonomy, systematics, species conservation, and resource development and utilization.

In this study, we generated the whole-chloroplast genomes of nine *Sophora* species representing three sections distributed in different habitats in China, and combined these data with five previously published *Sophora* plastomes to produce a comprehensive analysis of 14 species. Including genomic characters, contraction and expansion of IRs, repeats, identification of molecular markers, and phylogenetic inference. Our aims of this study were: i) to elucidate the molecular evolution and phylogenetic relationships in chloroplast genomes of *Sophora* species in the early-diverging legume subfamily Papilionoideae (Fabaceae); ii) to identify polymorphic loci for future phylogenetic inference of the genus *Sophora*; iii) to explore that the 23 kb inversion is present throughout the genus; and iv) to elucidation of the role of repeats in the identification of polymorphic loci.

## Materials and Methods

### Collection of Sample Materials, DNA Extraction, and Sequencing

Leaf materials of nine *Sophora* species were collected from the wild, dried, and kept in silica gel at the Herbarium of the Chengdu Institute of Biology (CDBI; Supplementary Table S1). Genomic DNA was extracted from silica-gel dried leaves using a modified cetyltrimethylammonium bromide (CTAB) method (Allen et al., 2006). The sheared low molecular weight DNA fragments were used to establish paired-end libraries according to the protocol of the Illumina manual (Illumina, San Diego, CA, USA). Completed libraries were pooled and sequenced using the Illumina NovaSeq 6,000 PE150 platform with 350 bp insert size (Berry Genomics, Beijing, China).

### Chloroplast Genome Assembly and Annotation

The Cleaned Illumina short reads were used to assemble the chloroplast genome using GetOrganelle v1.7.2 (Jin et al., 2020). Bandage (Wick et al., 2015) was then used to identify the circular maps to assess the quality of the assembly. Subsequently, the results were annotated using PGA (Qu et al., 2019) based on three reference genomes of *Sophora* from the NCBI: *Sophora alopecuroides* (NC_036102), *S. tonkinensis* Gagnep. (NC_042688), and *S. flavescens* Aiton (MH748034). Manual correction of genes with missing start and stop codons in annotations was performed using Geneious Prime 2021 (Biomatters Ltd., Auckland, New Zealand) and determination of whether pseudogenes are annotated. Finally, the linear chloroplast genome maps were visualized using OGDRAW v1.3.132 (Greiner et al., 2019).

### Comparative Genome Analysis and Molecular Marker Identification

The sequences of *Sophora alopecuroides* (NC_036102), *S. flavescens* (MH748034), *S. macrocarpa* Sm. (MT536779), *S. tonkinensis* (NC_042688), and *S. toromiro* Skottsb. (MT079958) were included in a comparative chloroplast genome analysis. The base content was determined with DNA Baser Sequence Assemble v5.15 (http://www.dnabaser.com/). To identify hypervariable regions, polymorphic sites, and nucleotide variability pi (π), the 14 chloroplast genome sequences were aligned using MAFFT v7.475 (Katoh and Standley, 2013) with default parameters. The pi (π) values were calculated though 600 bp sliding window with 200 bp steps available in DnaSP v5.10.1 (Librado and Rozas, 2009). Gene arrangements were further analyzed using Mauve alignment (Darling et al., 2004) with default parameters. The junction of the plastomes was analyzed using IRscope (Amiryousefi et al., 2018) to visualize the expansion and contraction of inverted repeats.

### Repeat Sequence Analysis

SSRs of 10 bp or more were detected using MISA (Beier et al., 2017) with the following parameters: mononucleotides, dinucleotides, trinucleotides, tetranucleotides, pentanucleotides, and hexanucleotides were set to 10, 5, 4, 3, 3, and 3, respectively. Tandem repeats were recognized using Tandem Repeats Finder v4.09 (Benson, 1999) with the following settings: the identity of repeats more than 90% were retained. Then the overlapped recurrences were removed manually. LSRs in the total genome, LSC, SSC and IR regions as well as forward, reverse, complement, and palindrome sequences were searched using REPuter (Kurtz et al., 2001) with the maximum repeat size set at 50 and the minimum at 30 (Hamming distance ≤3) between two repeats.

### Evolutionary and Phylogenetic Analysis

To reconstruct the phylogenetic relationships, we included the whole-chloroplast genome sequences and protein-coding sequences (CDS) of 30 plastomes from the subfamily Papilionoideae retrieved from the NCBI nucleotide database, and nine newly assembled *Sophora* plastid genomes (Supplementary Table S1). *Angylocalyx braunii* Harms (MN709877) and *Ateleia glazioveana* Baill. (MN709820) were used as the outgroup in the phylogenetic inference. All 39 whole plastid genome and CDS alignments were generated using MAFFT v7.450 (Katoh and Standley, 2013). Then Gblocks v0.9b (Talavera and Castresana, 2007) was used to filter the ambiguously aligned sites of two data matrices with default parameters. The nucleotide substitution models for the two data matrices were estimated using jModelTest v2.1.10 (Darriba et al., 2012) and the evolutionary best fit model was selected using the corrected Akaike Information Criterion (AICc). Phylogenetic trees were inferred using Maximum Likelihood (ML) and Bayesian Inference (BI). The ML analysis was performed using the IQ-TREE v1.4.241 (Nguyen et al., 2015) with branch support estimated using 2,000 replicates of both SH-like approximate likelihood-ratio test (SH-aLRT) and the ultrafast bootstrapping algorithm (UFboot; Guindon et al., 2010; Minh et al., 2013). The BI analysis was inferred using MrBayes v3.2.7a (Ronquist et al., 2012) and posterior probability was estimated with two independent Markov Chain Monte Carlo (MCMC) runs (20 million generations) with the preliminary 25% of sampled data discarded as burn-in. The resulted phylogenetic trees were visualized using Figtree v1.4.4 (https://github.com/rambaut/figtree/releases/tag/v1.4.4).

## Results

### Characteristics of the Newly Sequenced *Sophora* Plastomes

**Figure 1:**
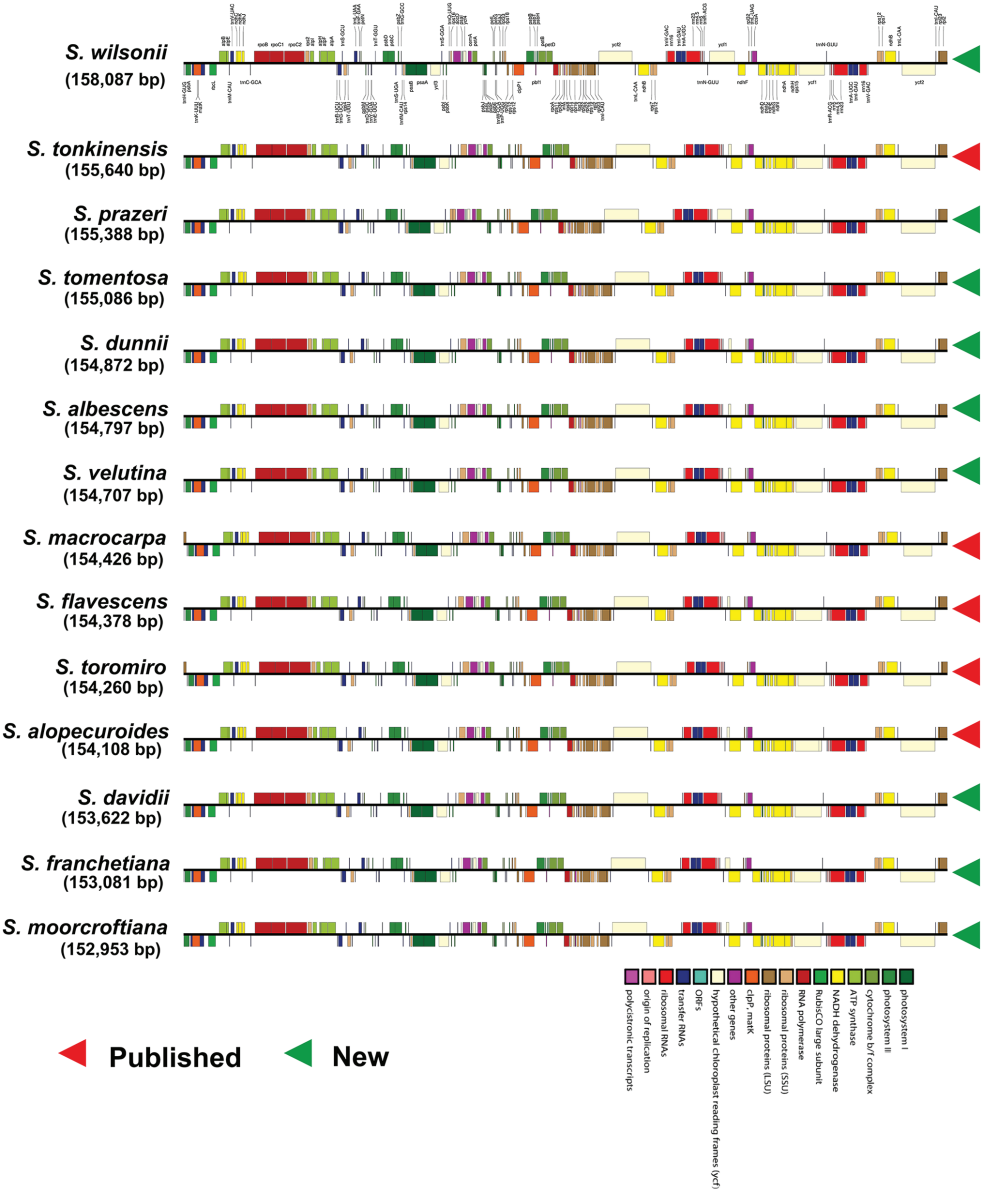
Complete linear plastomes maps of 14 *Sophora* species.

We obtained complete linear plastome maps (Figure 1) of *Sophora albescens* (Rehder) C.Y. Ma, *S. davidii* (Franch.) Skeels, *S. dunnii* Prain, *S. franchetiana* Dunn, *S. moorcroftiana* (Benth.) Benth. ex Baker, *S. prazeri* Prain, *S. tomentosa* L., *S. velutina* Lindl., and *S. wilsonii* Craib assemblies. These plastomes ranged from 152,953 bp (*S. moorcroftiana*) to 158,087 bp (*S. wilsonii*; Supplementary Table S2) and exhibited the typical quadripartite structure, including two IR regions of 25,800–30,609 bp separated by an LSC region of 83,138–85,127 bp and an SSC region of 13,466–18,342 bp (Supplementary Table S2). The GC content of the newly assembled plastomes ranged from 36.1% (*S. wilsonii*) to 36.7% (*S. davidii* and *S. moorcroftiana*; Supplementary Table S2). The GC content of the IR regions (40.3–42.9%) was high, whereas the LSC regions (33.8–34.3%) and SSC regions (29.5–30.6%) had lower GC content (Supplementary Figure S2B and Table S2). Nucleotide content of the IR, LSC, and SSC regions of each newly sequenced species are shown in Supplementary Figure S2A and Table S3.

The nine newly sequenced *Sophora* plastomes contained 129–130 genes, including 83–84 protein coding genes (seven duplicated in the IR region), 38 tRNA genes (seven duplicated in the IR region) and eight rRNA genes (four duplicated in the IR region), as shown in Supplementary Table S2 and S4. Fourteen genes contained one intron, including six tRNA genes (*trnK-UUU*, *trnV-UAC*, *trnG-UCC*, *trnL-UAA*, *trnI-GAU*, and *trnA-UGC*) and eight protein coding genes (*atpF*, *petB*, *petD*, *rpl16*, *rps16*, *rps12*, *rpl2*, and *ndhB*), while the other five protein coding genes (*rpoC1*, *clpP1*, *rps12*, *ycf3*, and *ndhA*) included two introns, and *ycf3* had three to four introns in some species (Supplementary Table S5). However, the *ndhA* gene of all newly-generated species, the *atpF* gene of *S. prazeri* and *S. wilsonii*, and the *rps16* gene of *S. prazeri* had two introns. In addition, the *rps16* gene was lost an intron in *S. franchetiana*, *S. moorcrofitiana* and *S.wilsonii*, while two introns were found in *S. prazeri*. The *rps12* gene had lost an intron in all *Sophora* species except for *S. albescens, S. dunnii*, and *S. velutina*.

### Comparative Genome Analysis

**Figure 2:**
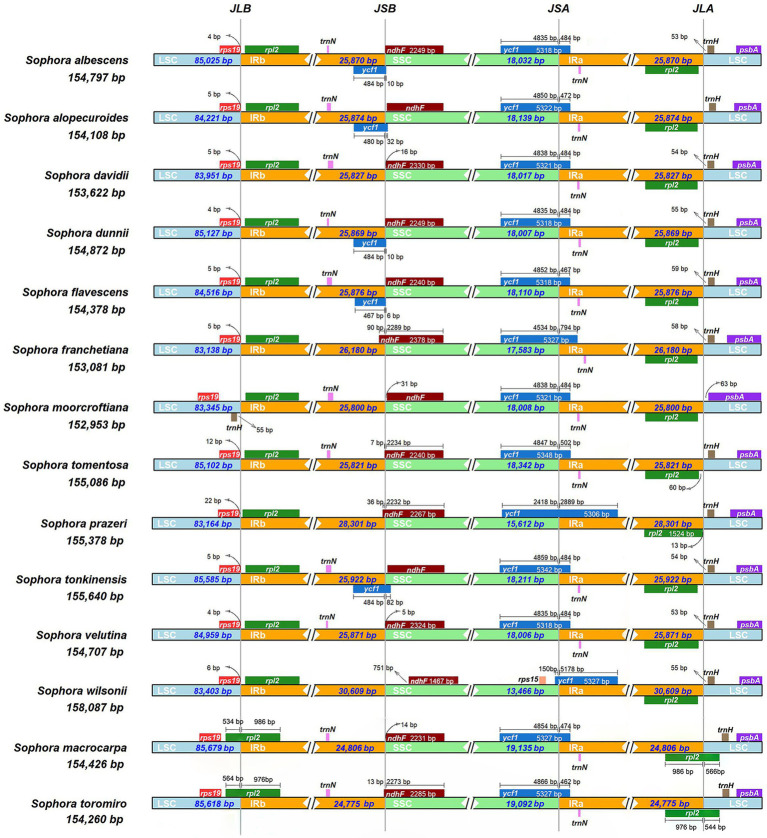
Analyses of expansion and contraction of inverted repeats in the 14 *Sophora* plastid genomes.

**Figure 3:**
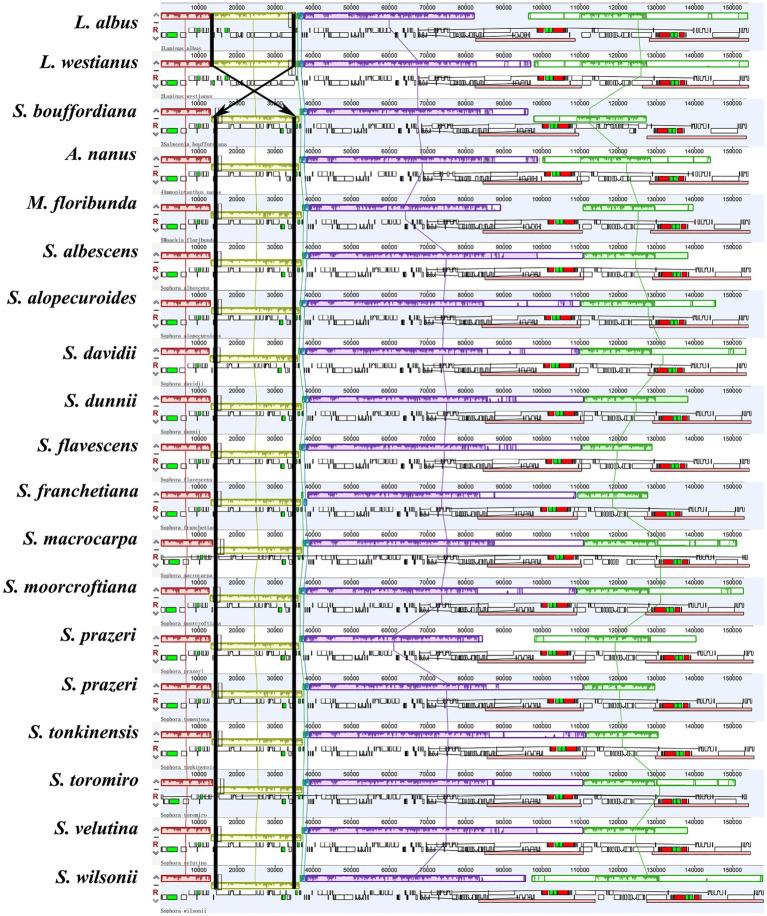
Genes arrangement using Mauve alignment in the 14 *Sophora* plastid genomes (White block: protein coding genes, black block: tRNA genes, green block: intron-containing tRNA genes, red block: rRNA genes).

We compared the JL (LSC/IR) and JS (IR/SSC) boundary positions of the *Sophora* species (Figure 2). The length of the IR regions ranges from 24,775–30,609 bp in 14 *Sophora* species with some expansion. The JLA (IRa-LSC: *rpl2* & *trnH*) and JLB (IRb-LSC: *rps19* & *rpl2*) boundaries showed high similarity in twelve *Sophora* species distributed in China. However, a notable difference had been found in *S. macrocarpa* and *S. toromiro* from Chile, where the gene *rpl2* crossed over the JLA and JLB boundaries and resulted in a pseudo-copy of *rpl2* due to the IR contraction. In *S. moorcrofitiana*, the gene *trnH* was relocated in LSC region near IRb regions, while in other 13 species the gene *trnH* was fully present within the LSC region near the IRa regions. At the IRa-SSC border, the *ycf1* gene crossed over the IRa-SSC border and extended into the IRa region ranging from 462 bp to 5,178 bp. In *S.prazeri* and *S. wilsonii*, the contraction of SSC resulted in the gene ycf1 was located in IRa region (2,889–5,178 bp). At the IRb-SSC border, the *ndhF* gene was fully present within the SSC region in *Sophora* species except *S. franchetiana*, *S. tomentosa*, *S. prazeri*, and *S. toromiro*, where the gene *ndhF* extended into the IRb regions with lengths ranging from 7 bp to 90 bp. One copy of the *ycf1* gene in the IRb region were lost or pseudogenized in this genus, except in *S. albescens*, *S. alopecuroides*, *S. dunnii*, *S. flavescens*, and *S. tonkinensis*. The gene order was conserved, except an approximately 23 kb inversion between *trnC-GCA* and *trnF-GAA* was observed in the LSC region (Figure 3 and Supplementary Table S2).

We compared the complete plastomes of 14 *Sophora* species and five other species with Mauve software. The results showed that the majority of the genes of this genus maintained a consistent position and orientation with no gene reversal detected, except for the reversal of the *trnH* gene in *S. moorcroftiana* appeared at the JLB boundary. In other words, the *trnH* gene was found in the first location in the LSC near the IRa region in *S. moorcrofitiana*, while in the other 13 species it was located in the LSC near the IRb region (Figure 3). The contraction and expansion of IR and subsequent generation of pseudogenes caused the total number of genes to vary across species from 129 to 130. We observed 17–18 duplicated genes in the IR, including 6–7 protein coding genes, seven tRNA genes, and four rRNA genes (Supplementary Table S2). Generation of pseudogenes of *ycf1* and *rpl2* was observed (Figure 2). In addition, a notable inversion was observed between *Lupinus* and other four genus (*Salweenia*, *Ammopipthus*, *Maackia* and *Sophora*) is shown in Figure 3.

### Repeat Sequences

**Figure 4:**
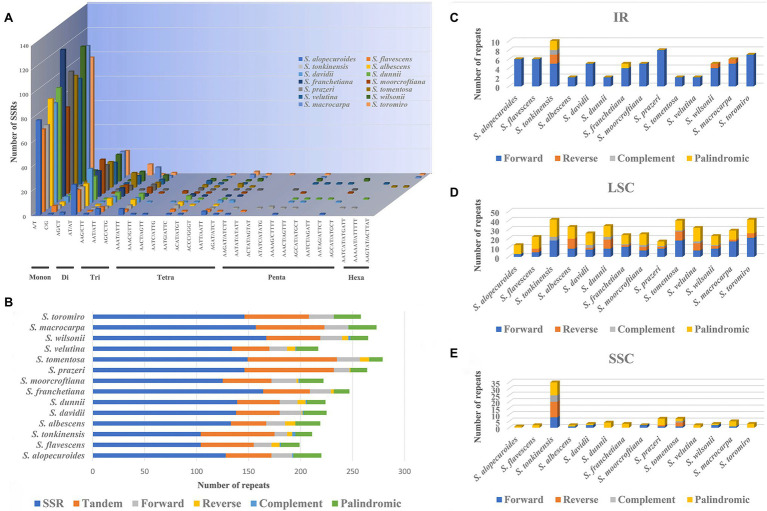
**(A)** Analysis of SSRs in 14 *Sophora* plastid genomes species; **(B)** Number of SSRs, Tandem, Forward, Reverse, Complement, and Palindromic repeats in 14 *Sophora* plastid genomes species; **(C-E)** Number of Forward, Reverse, Complement, and Palindromic repeats (IR, LSC, SSC) in 14 *Sophora* species.

The number of SSRs in *Sophora* species from 104 in *S. flavescens* and *S. tonkinensis* to 167 in *S. wilsonii* (Figures 4A,B and Supplementary Table S6). In our study, mononucleotide to tetranucleotide SSRs were found in all species of this genus. Pentanucleotide repeats were found in all species except *S. albescens*, *S. franchetiana*, and *S. tomentosa*. Hexanucleotides were only found in *S. tonkinensis*, *S. dunnii*, *S. tomentosa*, *S. velutina*, *S. macrocarpa*, and *S. toromiro* (Figure 4A; Supplementary Table S6). Among these SSRs, mononucleotide repeats were the most prevalent in Figures 4A,B. Only a small fraction consisted of dinucleotide, trinucleotide, tetranucleotide, pentanucleotide, or hexanucleotide repeat motifs (Figures 4A, B, and Supplementary Table S6).

LSRs in 14 *Sophora* plastomes were detected using REPter, with the maximum and minimum repeat size set to 50 and 30 (Hamming distance 1 to 3) between two repeats. A total of 373–628 repeats (Hamming distance 1 to 3), including forward, reverse, complement, and palindrome repeats were identified (Figure 4B and Supplementary Table S7). In general, forward and palindromic repeats were the most detected type detected in the *Sophora* plastomes, while complement repeats were the least common. The number of repeats in the IR, LSC, and SSC of 14 *Sophora* species are shown in Figures 4C-E; Supplementary Table S8. Results shown the most forward repeats were in the IR region, more forward and palindromic repeats in the LSC region, and about the same number in the SSC region in 14 *Sophora* species. *S. tonkinensis* had more repetitions in these three regions than other species. Besides, appropriately 763 tandem repeat sequences were identified in 14 *Sophora* plastomes (Figure 4B and Supplementary Table S7). The tandem repeats sequences ranged from 34 in *S. albescens* to 86 in *S. moorcrofitiana* and *S. prazeri*.

### Molecular Markers

**Figure 5:**
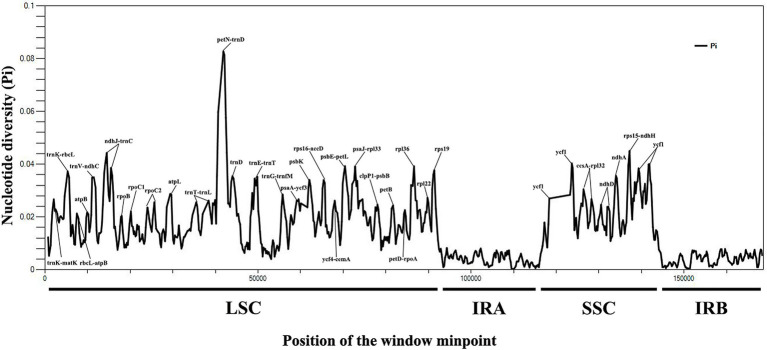
The nucleotide diversity (Pi) value (Y-axis) with their positions (X-axis) in each window of 14 *Sophora* species.

A total of 8,067 variable (polymorphic) sites were found in 169,235 nucleotide loci, including 4,145 singleton variable sites (SVS) and 3,922 parsimony informative sites (PIS). Three different categories under SVS were observed: 4,056 sites with two variants (SV2V), 88 sites with three variants (SV3V) and 1 site with four variants (SV4V). Similarly, PIS also has three categories: 3,638 sites with two variants (PIS2V), 272 sites with three variants (PIS3V) and 12 sites with four variants (PIS4V). The number of mutations and missing data of 14 *Sophora* species were 8,067, 25,232, respectively (Supplementary Table S9). We also calculated nucleotide variability pi (π) values for all 14 chloroplast genomes, ranging from 0 to 0.08275. The IR regions showed low nucleotide diversity (pi (π) < 0.008), indicating that most of the variation in the plastid genome of the 14 *Sophora* species occurred mainly in the LSC and SSC regions (Figure 5). The average nucleotide variations were the highest in the intergenic spacer (IGS) regions. The most divergent noncoding regions were *trnK-matK*, *trnK-rbcL*, *rbcL-atpB*, *atpB-trnM*, *trnV-ndhC*, *ndhJ-trnC*, *trnT-trnL*, *petN-trnD*, *trnE-trnT*, *trnG-trnfM*, *psaA-ycf3*, *rps16-accD*, *ycf4-cemA*, *psbE-petL*, *psaJ-rpl33*, *clpP1-psbB*, *petD-rpoA*, *rpl22-rps19*, *ccsA-rpl32*, and *rps15-ndhH* (pi (π) > 0.02; Supplementary Table S10). Although the coding regions were conserved in these plastid genomes, the protein coding regions of *petB*, *rpl36*, *ycf1*, *ndhD*, *ndhA*, *rpoB*, *ropC1*, *atpL*, and *psbK* were also included with high pi (π) values. Sequence variation was observed among the 14 genomes in *ndhJ-trnC*, *petN-trnD*, *trnE-trnT*, *psbE-petL*, *rpl22-rps19*, *ycf1*, and *ndhA* (> 200 bp; pi (π) > 0.035), which can be candidate barcode sequences. These molecular markers might be useful for future phylogenetic inference and population genetics studies of the genus *Sophora*.

### Phylogenetic Analysis

**Figure 6:**
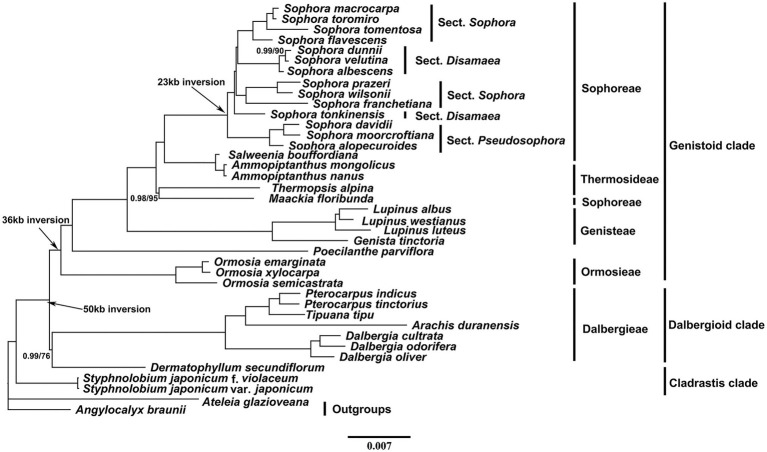
Phylogenetic tree obtained using the Bayesian Inference (BI) method of the plastid genomes of 39 taxa. Numbers above branches indicate Bayesian posterior probabilities (before slash) and ML bootstrap supports (after slash). The full support values are not indicated.

The phylogenies of the early-branching subfamily Papilionoideae inferred from the two data matrices (whole-chloroplast genome and CDS) and methods (ML and BI) yielded similar topologies (Figure 6 and Supplementary Figure S2, 3, 4). The best fit GTR model estimate of the complete chloroplast genomes and CDS were selected. The plastid phylogenomic analysis generated a strongly supported phylogeny with three distinct clades (*Cladrastis*, dalbergioid, and genistoid). Our phylogenetic analyses strong supported (BS = 100, and PP = 1.0) the monophyly of the *Sophora* genus, sect *Pseudosophora* and most lineages. However, the lineage consisting of sect. *Disamaea* and *Sophora* were separated into two monophyletic clades with full support, respectively. Based on the present study, the tribe Sophoreae is not monophyletic because species of the tribe Thermosideae clade was embedded in it with high support.

## Discussion

### Plastome Structure or Characteristics

The plastome structure, gene position and orientation, and gene content of the plastid genomes of *Sophora* species were highly conserved, as in other legume family species (Wang et al., 2018; Oyebanji et al., 2020; Zha et al., 2020; Zhang et al., 2020a). The plastomes revealed a typical circular tetrad structure, and no IR region was entirely lost, as had reported in *Pisum sativum* L. and *Medicago truncatula* Gaertn. (Saski et al., 2005). The plastomes of 14 *Sophora* species were 152,953–158,087 bp, with little variation in length between newly-generated and published genomes, indicating the homogeneity of the genus. There were 111–113 unique genes, including 76–78 protein coding genes, 31 tRNA and 4 rRNA genes. The total GC content of *Sophora* species was 36.1–36.7%, fell within the typical range for the plastomes of flowering plants (34–40%).

The expansion and contraction of the IR regions has been demonstrated to substantially contribute to the change in plastome size (Ruhlman and Jansen, 2014). The existence of IR expansion–contraction has been reported in various kinds of plants (Park et al., 2018; Xu and Wang, 2020; Guo et al., 2021). The expansion and contraction of the IR region is usually more variable in the LSC, while stable in the IR and SSC regions, except in *Corydalis* (Ma et al., 2013; Sun et al., 2013; Sun et al., 2016; Park et al., 2018; Xu and Wang, 2020). In *Sophora*, the IR regions of the plastomes started around the *rps19* gene, and terminated almost uniformly downstream of the *trnN*-GUU. The LSC, IR, and SSC sizes were relatively stable within the genus, except for *S. prazeri* and *S. wilsonii* which expanded markedly to about 5 kb. Although the gene order of the species was consistent, the nine newly reported genomes in this research shared a ~ 23 kb inversion spanning *trnC*-*GCA* to *trnF*-*GAA* in the LSC region, which is similar to that first reported in *S. alopecuroides* (Zha et al., 2020). An approximately 23 kb inversion was observed and counted in *Salweenia* (22, 608 bp), *Maackia* (23,338 bp), *Thermopsis* (23, 601 bp), and *Ammopipanthus* (22,563–22,564 bp) in this study. The inversion therefore occurred prior to the divergence of tribe Sophoreae and Thermopsideae.

Gene and intron content are variable relative to most plants (Guisinger et al., 2011). We detected the loss of the *trnT-CGU* in all mainland’s species compared with two published island species (*S. macrocarpa* and *S. toromiro*; Pezoa et al., 2021). Introns, particularly those located in specific regions, are important for the functionality and regulation of gene expression (Xu et al., 2008). In the present study, except in morphological confusing species *S. albescens*, *S. dunnii*, and *S. velutina*, the *rps12* gene lost an intron in this genus which was specific to Desmodieae (Jin et al., 2019). We also identified the addition of an intron in *ndhA*, *atpF*, *rps16*, and *rpoC1*, a finding which differs from those of previous studies (Li et al., 2021; Oyebanji et al., 2020; Zhao et al., 2020; Guo et al., 2021). Compared to the two species (*S. macrocarpa* and *S. toromiro*) distributed in Chile, the *ycf3* gene was increased by one to two introns and the gene *rrn23* lost introns within each of the taxa distributed in China.

### Repeated Sequences

SSRs are extensively distributed in the chloroplast genomes of eukaryotes, and their structural simplicity, relative conservation, and polymorphism make them valuable molecular markers that are broadly used for species identification, population genetics, and polymorphism research (Pauwels et al., 2012). Appropriately 104–167 SSRs were identified in the plastid genome of *Sophora* species, together with mononucleotides, dinucleotides, tetranucleotides, trinucleotides, pentanucleotides and hexanucleotides. Among them, mononucleotide nucleotides rich in A/T were the most abundant in 14 species. AT/AT repeats, AAT/AAT repeats, and AAAT/AAAT repeats were prevalent in all species (Figure 3A). This phenomenon may occur because the A/T variation occurs more easily than the G/C mutation (Li et al., 2021). Similar cases has been reported in previous study in which SSRs generally consist of polyA or polyT repeats, and rarely contain G or C repeats (Zha et al., 2020). These newly detected SSRs will be useful for the development of genetic markers for the Sophora species in future studies.

Large repeat sequences are informative for phylogenetic studies of *Sophora* species and play a crucial role in plastomes evolution which have been suggested as a proxy to identify mutational hotspots in various angiosperm species (Abdullah, Mehmood, et al., 2020b, 2021b; Abdullah, Henriquez, et al., 2021b). In this study, we identified 10 highly polymorphic loci (pi (π) > 0.035) suitable for inferring the phylogeny of *Sophora* species. Among these, three loci (*ndhJ*-*trnC*, *ndhA* and *ycf1*) belong to the regions where repeats are present, which showed the highest incidence of polymorphisms (Supplementary Table S11). Here, our findings support the use of repeats as a proxy, and this approach may also be helpful for the identification of suitable polymorphic loci for phylogenetic inference of other taxonomically complex genera.

### Identification of Molecular Markers

DNA barcoding technology is widely used in studies for the species identification, phylogeny, and evolution (Liu et al., 2019b). Suitable polymorphic regions need to be identified if previous studies have been unable to resolve taxonomic issues and the phylogeny of the genus with low polymorphism of molecular markers (Abdullah, Mehmood, et al., 2021b). Comparative genomic analysis showed that the DNA sequences of *Sophora* species were relatively conserved in the IR region, and had relatively large number of variations in the LSC and SSC. This may be caused by gene conversion or loss between the two IR regions and the LSC and SSC boundaries (Khakhlova and Bock, 2006; Li et al., 2016). Strategies to search for potential DNA barcodes have been reported to be successfully applied to various angiosperm species (Liu et al., 2019a). In this study, we identified 10 polymorphic regions by comparison of 14 *Sophora* species using sliding window analysis. The genes *ndhJ*-*trnC*, *petN*-*trnD*, *trnE*-*trnT*, *psbE*-*petL*, *rpl22*-*rps19*, and protein coding regions of *ycf1* and *ndhA* (pi (π) > 0.035) can be as a candidate barcode sequences. The genes *ycf1* and *ndhA* were also reported in the subfamily Dialioideae (Fabaceae), which had the highest nucleotide diversity among all protein coding genes (Bai et al., 2021). To evaluate the ability of selected sequences to identify in *Sophora* species, we constructed the ML and BI tree based on seven concatenated markers with a tree topology similar to that whole-chloroplast genomes and CDS (Figure 6 and Supplementary Figure S5). Therefore, these molecular markers might be useful for phylogenetic and population genetic studies of the genus *Sophora*.

### Phylogenetic Relationships

The plastid phylogenomic analysis generated a strongly supported phylogeny with three distinct clades (*Cladrastis*, dalbergioid, and genistoid), which is consistent with the results of earlier studies (Martin et al., 2014; Choi and Choi, 2017; Liu et al., 2019a; Wei et al., 2020; Win et al., 2020; Zha et al., 2020; Zhang et al., 2020a). All *Sophora* lineages were well-supported in the phylogenetic tree, indicating that the implementation of complete plastome data-set has the potential to resolve the phylogenetic relationships of the genus, which could be a guidance to resolve the taxonomic controversy of the genus. Our findings support the clustering of *S. davidii*, *S. moorcroftiana*, and *S. alopecuroides* as a monophyletic clade (sect. *Pseudosophora*), as the first diverged section of this genus was resolved with high support values. However, the morphological monophyly of sect. *Sophora* and sect. *Disamaea* was not supported compared with morphological evidence from previous sectional divisions (Tsoong and Ma, 1981; Ma, 1990). In sect. *Disamaea*, the considerable morphological differences between *S. tonkinensis* (e.g., yellow corolla, leathery leaf blade, near-absence of stipules, etc.) and the other three species (*S. albescens*, *S. dunnii* and *S. velutina*), which were divided into two branches consistent with their different habitat distribution (karst landforms vs. arid-hot valley). In sect. *Sophora*, three morphologically similar species, *S. prazeri*, *S. wilsonii*, and *S. franchetiana*, clustered into one clade and *S. tomentosa*, *S. macrocarpa*, *S. toromiro*, and *S. flavescens* clustered into another clade. Also, *S. tomentosa* is sister to the *S. macrocarpa* and *S. toromiro* clade, an observation which is consistent with the results based on ITS and *rbcL* evidence (Mitchell and Heenan, 2002; Heenan et al., 2004). Therefore, we propose to include more material and evidence in future studies to establish a clearer phylogeny of sect. *Disamaea* as well as sect. *Sophora*.

Ma (1990) indicated that *Ormosia* belongs to the first diverged taxa of the tribe Sophoreae and *Sophora* belongs to recently diverged lineage of the tribe based on morphological characters. However, our results showed that *Sophora* is more closely related to the genera *Salweenia* and *Amopiptanthus* than to *Ormosia* and *Stypholobium*, which is consistent with previous studies (Win et al., 2020; Wei et al., 2020; Zha et al., 2020; Zhang et al., 2020a). In the Genitoid, the monophyly of tribes Ormosieae and Genisteae are well-supported, whereas the tribe Thermopsideae is embedded in the tribe Sophoreae, which is consistent with previous studies (Choi and Choi, 2017). That is, *Salweenia* and *Ammopiptanthus* cluster into one clade, while *Maackia* and *Thermopsis* cluster into another clade, resulting in the tribe Sophoreae not being monophyletic. Besides, the plastid phylogeny might only resolve an evolutionary line of matrilineal inheritance, and does not necessarily reflecting the full coalescent history (Wang et al., 2018). Thus, more material and evidence will be needed from future studies which will provide a higher resolution of the relationships among genera in this subfamily, possibly through more intensive sampling of taxa, combined with data from other genomic compartments.

## Conclusion

In this study, we assembled nine plastid genomes and provided insights into the plastome structure of *Sophora* species, which will provide a basis for inferring species trees and exploring non-divergent relationships. Comparative genome analysis showed that this genus exhibited extensive rearrangements, including gene losses, gene duplications, relocations, ~23 kb inversions, IR expansions, and pseudogenizations. Meanwhile, the plastid data-set proved new insights in the resolution of the phylogeny of *Sophora*, and it may be used to construct a robust phylogeny for *Sophora* in further studies. The findings obtained in this work will be valuable for further studies on the taxonomy, phylogeny, and evolution of *Sophora*, a taxonomically problematic but challenging genus.

## Data Availability Statement

The chloroplast genome sequences of *Sophora* species were submitted on the National Center for Biotechnology Information (NCBI) and the accession numbers were: MW940392–MW940400.

## Author Contributions

ML, XG, and BX conceived and designed the study. HD, ML, and JZ collected the sample. JZ and ML analyzed the data. ML wrote the manuscript. XG and BX revised the paper. All authors contributed to the article and approved the submitted version.

## Funding

This work was supported by the National Natural Science Foundation of China (Grant #31570196), China-Croatia “Belt and Road” Joint Laboratory on Biodiversity and Ecosystem Services (Grant No. 2020YFE0203200), and the Second Tibetan Plateau Scientific Expedition and Research (STEP) program (Grant No. 2019QZKK0502).

## Conflict of Interest

The authors declare that the research was conducted in the absence of any commercial or financial relationships that could be construed as a potential conflict of interest.

## Publisher’s Note

All claims expressed in this article are solely those of the authors and do not necessarily represent those of their affiliated organizations, or those of the publisher, the editors and the reviewers. Any product that may be evaluated in this article, or claim that may be made by its manufacturer, is not guaranteed or endorsed by the publisher.

## References

[ref3] Abdullah Henriquez C. L. Croat T. B. Poczai P. Ahmed I. (2021a). Mutational dynamics of aroid chloroplast genomes II. Front. Genet. 11, 1562. doi: 10.3389/fgene.2020.610838, PMID: 33552129 PMC7854696

[ref2] Abdullah Henriquez C. L. Mehmood F. Carlsen M. M. Islam M. Waheed M.. (2020a). Complete chloroplast genomes of Anthurium huixtlense and Pothos scandens (Pothoideae, Araceae): unique inverted repeat expansion and contraction affect rate of evolution. J. Mol. Evol. 88, 562–574. doi: 10.1007/s00239-020-09958-w, PMID: 32642873 PMC7445159

[ref5] Abdullah Mehmood, F., Rahim, A., Heidari, P., Ahmed, I., and Poczai., P. (2021b). Comparative plastome analysis of Blumea, with implications for genome evolution and phylogeny of Asteroideae. Ecol. Evol. 11, 7810–7826. doi: 10.1002/ece3.7614, PMID: 34188853 PMC8216946

[ref1] Abdullah Mehmood F. Shahzadi I. Ali Z. Islam M. Naeem M.. (2020b). Correlations among oligonucleotide repeats, nucleotide substitutions and insertion-deletion mutations in chloroplast genomes of plant family Malvaceae. J. Syst. Evol. 59, 388–402. doi: 10.1111/jse.12585

[ref4] Abdullah Shahzadi I. Mehmood F. Ali Z. Malik M. S. Waseem S.. (2019). Comparative analyses of chloroplast genomes among three Firmiana species: identification of mutational hotspots and phylogenetic relationship with other species of Malvaceae. Plant Gene 19, 100199. doi: 10.1016/j.plgene.2019.100199

[ref6] Allen G., Flores-Vergara, M., Krasynanski S. Kumar S. Thompson W. F. (2006). A modified protocol for rapid DNA isolation from plant tissues using cetyltrimethylammonium bromide. Nat. Protoc. 1, 2320–2325. doi: 10.1038/nprot.2006.384, PMID: 17406474

[ref7] Alqahtani A. A. Jansen R. K. (2021). The evolutionary fate of *rpl32* and *rps16* losses in the *Euphorbia schimperi* (Euphorbiaceae) plastome. Sci. Rep. 11, 7466. doi: 10.1038/s41598-021-86820-z, PMID: 33811236 PMC8018952

[ref8] Amiryousefi A. Hyvönen J. Poczai P. (2018). IRscope: an online program to visualize the junction sites of chloroplast genomes. Bioinformatics 34, 3030–3031. doi: 10.1093/bioinformatics/bty220, PMID: 29659705

[ref9] Asaf S. Khan A. Lubna K. G., Lee, I. J., and Al-Harrasi, A. (2020). Expanded inverted repeat region with large scale inversion in the first complete plastid genome sequence of *Plantago ovata*. Sci. Rep. 10, 3881. doi: 10.1038/s41598-020-60803-y, PMID: 32127603 PMC7054531

[ref10] Bai H. R. Oyebanji O. Zhang R. Yi T. S. (2021). Plastid phylogenomic insights into the evolution of subfamily Dialioideae (Leguminosae). Plant Diversity 43, 27–34. doi: 10.1016/j.pld.2020.06.008, PMID: 33778222 PMC7987570

[ref11] Beier S. Thiel T. Muench T. Scholz U. Mascher M. (2017). MISA–web: a web server for microsatellite prediction. Bioinformatics 33, 2583–2585. doi: 10.1093/bioinformatics/btx198, PMID: 28398459 PMC5870701

[ref12] Benson G. (1999). Tandem repeats finder: a program to analyze DNA sequences. Nucleic Acids Res. 27, 573–580. doi: 10.1093/nar/27.2.573, PMID: 9862982 PMC148217

[ref13] Burke S. V. Lin C. S. Wysocki W. P. Clark L. Duvall M. (2016). Phylogenomics and plastome evolution of tropical forest grasses (*Leptaspis, Streptochaeta*: Poaceae). Front. Plant Sci. 7, 1993. doi: 10.3389/fpls.2016.01993, PMID: 28083012 PMC5186769

[ref14] Cai Z. Guisinger M. M. Kim H. Ruck E. C. Blazier J. C. Mcmurtry V.. (2008). Extensive reorganization of the plastid genome of *Trifolium subterraneum* (Fabaceae) is associated with numerous repeated sequences and novel DNA insertions. J. Mol. Evol. 67, 696–704. doi: 10.1007/s00239-008-9180-7, PMID: 19018585

[ref15] Chen M. Ding Y. Tong Z. Q. (2020). Efficacy and safety of Sophora flavescens (Kushen) based traditional chinese medicine in the treatment of ulcerative colitis: clinical evidence and potential mechanisms. Front. Pharmacol. 11:603476. doi: 10.3389/fphar.2020.603476, PMID: 33362558 PMC7758483

[ref16] Choi I. S. Choi B. (2017). The distinct plastid genome structure of Maackia fauriei (Fabaceae: Papilionoideae) and its systematic implications for genistoids and tribe Tr. Sophoreae. PLoS One 12:e0173766. doi: 10.1371/journal.pone.0173766, PMID: 28399123 PMC5388331

[ref17] Darling A. C. E. Mau B. Blattner F. R. Perna N. T. (2004). Mauve: multiple alignment of conserved genomic sequence with rearrangements. Genome Res. 14, 1394–1403. doi: 10.1101/gr.2289704, PMID: 15231754 PMC442156

[ref18] Darriba D. Taboada G. L. Doallo R. Posada D. (2012). jModelTest 2: more models, new heuristics and parallel computing. Nat. Methods 9, 772–772. doi: 10.1038/nmeth.2109, PMID: 22847109 PMC4594756

[ref19] Doyle J. Doyle J. L. Ballenger J. Palmer J. (1996). The distribution and phylogenetic significance of a 50–kb chloroplast DNA inversion in the flowering plant family Leguminosae. Mol. Phylogenet. Evol. 5, 429–438. doi: 10.1006/mpev.1996.0038, PMID: 8728401

[ref20] Doyle J. Doyle J. L. Palmer J. (1995). Multiple independent losses of two genes and one intron from legume chloroplast genomes. Syst. Bot. 20, 272. doi: 10.2307/2419496

[ref21] Duan L. Harris A. Ye W. Deng S. Song Z. Chen H. F.. (2019). Untangling the taxonomy of the *Cladrastis* clade (Leguminosae: Papilionoideae) by integrating phylogenetics and ecological evidence. Taxon 68, 1189–1203. doi: 10.1002/tax.12155

[ref22] Dugas D. V. Hernandez D. Koenen E. Schwarz E. N. Straub S. Hughes C.. (2015). Mimosoid legume plastome evolution: IR expansion, tandem repeat expansions, and accelerated rate of evolution in clpP. Sci. Rep. 5, 16958. doi: 10.1038/srep16958, PMID: 26592928 PMC4655330

[ref23] Feng L. Gu L. F. Luo J. Fu A. Ding Q. Yiu S.. (2017). Complete plastid genomes of the genus *Ammopiptanthus* and identification of a novel 23–kb rearrangement. Conserv. Genet. Resour. 9, 647–650. doi: 10.1007/s12686-017-0747-8

[ref24] Fonseca L. H. M. Lohmann L. (2017). Plastome rearrangements in the “*Adenocalymma*-*Neojobertia*” clade (Bignonieae, Bignoniaceae) and its phylogenetic implications. Front. Plant Sci. 8, 1875. doi: 10.3389/fpls.2017.01875, PMID: 29163600 PMC5672021

[ref25] Greiner S. Lehwark P. Bock R. (2019). OrganellarGenomeDRAW (OGDRAW) version 1.3.1: expanded toolkit for the graphical visualization of organellar genomes. Nucleic Acids Res. 47, W59–W64. doi: 10.1093/nar/gkz238, PMID: 30949694 PMC6602502

[ref26] Guindon S. Dufayard J. F. Lefort V. Anisimova M. Hordijk W. Gascuel O. (2010). New algorithms and methods to estimate maximum-likelihood phylogenies: assessing the performance of PhyML 3.0. Syst. Biol. 59, 307–321. doi: 10.1093/sysbio/syq010, PMID: 20525638

[ref27] Guisinger M. M. Kuehl J. V. Boore J. L. Jasen R. K. (2011). Extreme reconfiguration of plastid genomes in the angiosperm family Geraniaceae: rearrangements, repeats, and codon usage. Mol. Biol. Evol. 28(1), 583–600. doi: 10.1093/molbev/msq229, PMID: 20805190

[ref28] Guo Y. Y. Yang J. X. Li H. Zhao H. (2021). Chloroplast genomes of two species of *Cypripedium*: expanded genome size and proliferation of AT–biased repeat sequences. Front. Plant Sci. 12:609729. doi: 10.3389/fpls.2021.609729, PMID: 33633763 PMC7900419

[ref29] Haberle R. C. Fourcade H. Boore J. Jansen R. (2008). Extensive rearrangements in the chloroplast genome of *Trachelium caeruleum* are associated with repeats and tRNA genes. J. Mol. Evol. 66, 350–361. doi: 10.1007/s00239-008-9086-4, PMID: 18330485

[ref30] Heenan P. Dawson M. Wagstaff S. (2004). The relationship of *Sophora sect. Edwardsia* (Fabaceae) to *Sophora tomentosa*, the type species of the genus Sophora, observed from DNA sequence data and morphological characters. Bot. J. Linn. Soc. 146, 439–446. doi: 10.1111/j.1095-8339.2004.00348.x

[ref31] Hurr K. Lockhart P. Heenan P. Penny D. (1999). Evidence for the recent dispersal of *Sophora* (Leguminosae) around the southern oceans: molecular data. J. Biogeogr. 26, 565–577. doi: 10.1046/j.1365-2699.1999.00302.x

[ref32] Iinuma M. Ohyama M. Tanaka T. (1995). Six flavonostilbenes and a flavanone in roots of *Sophora alopecuroides*. Phytochemistry 38, 519–525. doi: 10.1016/0031-9422(94)00720-E

[ref33] Jansen R. Wojciechowski M. Sanniyasi E. Lee S. Daniell H. (2008). Complete plastid genome sequence of the chickpea (*Cicer arietinum*) and the phylogenetic distribution of *rps12* and *clpP* intron losses among legumes (Leguminosae). Mol. Phylogenet. Evol. 48, 1204–1217. doi: 10.1016/j.ympev.2008.06.013, PMID: 18638561 PMC2586962

[ref34] Jin J. Yu W. B. Yang J. Song Y. Depamphilis C. Yi T. S.. (2020). GetOrganelle: a fast and versatile toolkit for accurate de novo assembly of organelle genomes. Genome Biol. 21, 241. doi: 10.1186/s13059-020-02154-5, PMID: 32912315 PMC7488116

[ref35] Jin D. P. Choi I. S. Choi B. H. (2019). Plastid genome evolution in tribe Desmodieae (Fabaceae: Papilionoideae). PLoS One 14(6):e0218743. doi: 10.1371/journal.pone.0218743, PMID: 31233545 PMC6590825

[ref36] Katoh K. Standley D. (2013). MAFFT multiple sequence alignment software version 7: improvements in performance and usability. Mol. Biol. Evol. 30, 772–780. doi: 10.1093/molbev/mst010, PMID: 23329690 PMC3603318

[ref37] Keller J., Rousseau–Gueutin, M., Martin G. Morice J. Boutte J. Coissac E.. (2017). The evolutionary fate of the chloroplast and nuclear *rps16* genes as revealed through the sequencing and comparative analyses of four novel legume chloroplast genomes from *Lupinus*. DNA Res. 24, 343–358. doi: 10.1093/dnares/dsx006, PMID: 28338826 PMC5737547

[ref38] Khakhlova O. Bock R. (2006). Elimination of deleterious mutations in plastid genomes by gene conversion. The Plant journal: for Cell and Molecular Biology 46, 85–94. doi: 10.1111/j.1365-313X.2006.02673.x, PMID: 16553897

[ref39] Kim K. Choi K. S. Jansen R. (2005). Two chloroplast DNA inversions originated simultaneously during the early evolution of the sunflower family (Asteraceae). Mol. Biol. Evol. 22, 1783–1792. doi: 10.1093/molbev/msi174, PMID: 15917497

[ref40] Kurtz S. Choudhuri J. V. Ohlebusch E. Schleiermacher C. Stoye J. Giegerich R. (2001). REPuter: the manifold applications of repeat analysis on a genomic scale. Nucleic Acids Res. 29, 4633–4642. doi: 10.1093/nar/29.22.4633, PMID: 11713313 PMC92531

[ref41] Lavin M. Doyle J. Palmer J. (1990). Evolutionary significance of the loss of the chloroplast-DNA inverted repeat in the Leguminosae subfamily Papilionoideae. Evolution 44, 390–402. doi: 10.2307/2409416, PMID: 28564377

[ref42] Lee C. Ruhlman T. A. Jansen R. (2020). Unprecedented intraindividual structural heteroplasmy in eleocharis (Cyperaceae, Poales) plastomes. Genome Biol. Evol. 12, 641–655. doi: 10.1093/gbe/evaa076, PMID: 32282915 PMC7426004

[ref43] Lee H. L. Jansen R. Chumley T. W. Kim K. (2007). Gene relocations within chloroplast genomes of *Jasminum and Menodora* (Oleaceae) are due to multiple, overlapping inversions. Mol. Biol. Evol. 24, 1161–1180. doi: 10.1093/molbev/msm036, PMID: 17329229

[ref44] Li F. W. Kuo L. Y. Pryer K. M. Rothfels C. J. (2016). Genes translocated into the plastid inverted repeat show decelerated substitution rates and elevated GC content. Genome Biol. Evol. 8, 2452–2458. doi: 10.1093/gbe/evw167, PMID: 27401175 PMC5010901

[ref45] Li Y. T. Dong Y. Liu Y. C. Yu X. Y. Yang M. S. Huang Y. R. (2021). Comparative analyses of *Euonymus* chloroplast genomes: genetic structure, screening for loci with suitable polymorphism, positive selection genes, and phylogenetic relationships within Celastrineae. Front. Plant Sci. 11:593984. doi: 10.3389/fpls.2020.593984, PMID: 33643327 PMC7905392

[ref46] Liang L. Wang X. Zhang X. Ji B. Yan H. C. Deng H.. (2012). Sophoridine exerts an anti–colorectal carcinoma effect through apoptosis induction in vitro and in vivo. Life Sci. 91, 1295–1303. doi: 10.1016/j.lfs.2012.09.021, PMID: 23069582

[ref47] Librado P. Rozas J. (2009). Dna SP v5: a software for comprehensive analysis of DNA polymorphism data. Bioinformatics 25, 1451–1452. doi: 10.1093/bioinformatics/btp187, PMID: 19346325

[ref48] Liu H. Su Z. Yu S. Liu J. Yin X. Zhang G.. (2019a). Genome comparison reveals mutation hotspots in the chloroplast genome and phylogenetic relationships of *Ormosia* species. Bio Med Research International 2019. doi: 10.1155/2019/7265030, PMID: 31531364 PMC6720362

[ref49] Liu Q. Li X. Li M. Xu W. Schwarzacher T., and Heslop-Harrison, J. S. (2020). Comparative chloroplast genome analyses of *Avena*: insights into evolutionary dynamics and phylogeny. BMC Plant Biol. 20, 406. doi: 10.1186/s12870-020-02621-y, PMID: 32878602 PMC7466839

[ref50] Liu X. Chang E. Liu J. Huang Y. Wang Y. Yao N.. (2019b). Complete chloroplast genome sequence and phylogenetic analysis of *Quercus bawanglingensis* Huang, Li et Xing, a vulnerable oak tree in China. Forests 10, 587. doi: 10.3390/ijms19082443

[ref51] Ma C. Y. (1990). Review of the classification system on the genus *Sophora*. Acta Phytotaxonomica Sinica 10, 77–86.

[ref52] Ma J. Yang B. Zhu W. Sun L. Tian J. Wang X. (2013). The complete chloroplast genome sequence of *Mahonia bealei* (Berberidaceae) reveals a significant expansion of the inverted repeat and phylogenetic relationship with other angiosperms. Gene 528, 120–131. doi: 10.1016/j.gene.2013.07.037, PMID: 23900198

[ref53] Martin G., Rousseau–Gueutin, M., Cordonnier S. Lima O., Michon–Coudouel, S., Naquin D.. (2014). The first complete chloroplast genome of the Genistoid legume *Lupinus luteus*: evidence for a novel major lineage–specific rearrangement and new insights regarding plastome evolution in the legume family. Ann. Bot. 113, 1197–1210. doi: 10.1093/aob/mcu050, PMID: 24769537 PMC4030815

[ref54] Mattapha S. Suddee S. Rueangruea S. (2018). *Sophora huamotensis*, a new species of *Sophora* (Fabaceae-Papilionoideae-Sophoreae) from Thailand. Thai Forest Bulletin (Botany) 46, 4–9. doi: 10.20531/tfb.2018.46.1.02

[ref55] Minh B. Q. Nguyen M. A. T. Von-Haeseler A. (2013). Ultrafast approximation for phylogenetic bootstrap. Mol. Biol. Evol. 30, 1188–1195. doi: 10.1093/molbev/mst024, PMID: 23418397 PMC3670741

[ref56] Mitchell A. Heenan P. (2002). *Sophora sect. Edwardsia* (Fabaceae): further evidence from nrDNA sequence data of a recent and rapid radiation around the southern oceans. Bot. J. Linn. Soc. 140, 435–441. doi: 10.1046/j.1095-8339.2002.00101.x

[ref57] Mower J. P. Vickrey T. L. (2018). Structural diversity among plastid genomes of land plants. Advance in Botanical Research 85, 263–292. doi: 10.1016/bs.abr.2017.11.013

[ref58] Nguyen L. T. Schmidt H. Haeseler A.V. Minh B. (2015). IQ–TREE: A fast and effective stochastic algorithm for estimating Maximum–Likelihood phylogenies. Mol. Biol. Evol. 32, 268–274. doi: 10.1093/molbev/msu300, IQ-TREE: a fast and effective stochastic algorithm for estimating maximum-likelihood phylogenies, PMID: 25371430 PMC4271533

[ref59] Oyebanji O. Zhang R. Chen S. Y. Yi T. S. (2020). New insights into the plastome evolution of the Millettioid/Phaseoloid clade (Papilionoideae, Leguminosae). Front. Plant Sci. 11, 151. doi: 10.3389/fpls.2020.00151, PMID: 32210983 PMC7076112

[ref60] Palmer J. (1985). Comparative organization of chloroplast genomes. Annu. Rev. Genet. 19, 325–354. doi: 10.1146/annurev.ge.19.120185.001545, PMID: 3936406

[ref61] Park S. An B. Park S. (2018). Reconfiguration of the plastid genome in *Lamprocapnos spectabilis*: IR boundary shifting, inversion, and intraspecific variation. Sci. Rep. 8, 13568. doi: 10.1038/s41598-018-31938-w, PMID: 30206286 PMC6134119

[ref62] Pauwels M. Vekemans X. Godé C. Frérot H. Castric V., and Saumitou-Laprade, P. (2012). Nuclear and chloroplast DNA phylogeography reveals vicariance among European populations of the model species for the study of metal tolerance, *Arabidopsis halleri* (Brassicaceae). New Phytol. 193, 916–928. doi: 10.1111/j.1469-8137.2011.04003.x 22225532

[ref63] Pennington R. T. Stirton C. H. Schrire B. D. (2005). “Tr. Sophoreae.” in Legumes of the World. eds. Lewis G. P. Schrire B. D. Mackinder B. A. Lock M. (Royal Botanic Gardens, Kew) 227–249.

[ref64] Pezoa I. Villacreses J. Rubilar M. Pizarro C. Galleguillos M. J. Ejimentewica T.. (2021). Generation of chloroplast molecular markers to differentiate *Sophora toromiro* and its hybrids as a first approach to its reintroduction in Rapa Nui (Easter Island). Plan. Theory 10(2), 342. doi: 10.3390/plants10020342, PMID: 33578941 PMC7916652

[ref65] Qu X. J. Moore M. Li D. Yi T. S. (2019). PGA: a software package for rapid, accurate, and flexible batch annotation of plastomes. Plant Methods 15, 50. doi: 10.1186/s13007-019-0435-7, PMID: 31139240 PMC6528300

[ref66] Ronquist F. Teslenk M. Van der Mark P. Ayres D. Darling A. Höhna S.. (2012). MrBayes 3.2: efficient Bayesian phylogenetic inference and model choice across a large model space. Syst. Biol. 61, 539–542. doi: 10.1093/sysbio/sys029, PMID: 22357727 PMC3329765

[ref67] Röschenbleck J. Wicke S. Weinl S. Kudla J. Müller K. (2017). Genus–wide screening reveals four distinct types of structural plastid genome organization in *Pelargonium* (Geraniaceae). Genome Biol. Evol. 9, 64–76. doi: 10.1093/gbe/evw271, PMID: 28172771 PMC5381562

[ref68] Ruhlman T. A. Jansen R. (2014). The plastid genomes of flowering plants. Methods Mol. Biol. 1132, 3–38. doi: 10.1007/978-1-62703-995-6_1 24599844

[ref69] Sablok G. Amiryousefi A. He X. Hyvönen J. Poczai P. (2019). Sequencing the plastid genome of giant ragweed (*Ambrosia trifida*, Asteraceae) from a herbarium specimen. Front. Plant Sci. 10, 218. doi: 10.3389/fpls.2019.00218, PMID: 30873197 PMC6403193

[ref70] Saski C. Lee S. Daniell H. Wood T. Tomkins J. Kim H.. (2005). Complete chloroplast genome sequence of *Glycine max* and comparative analyses with other legume genomes. Plant Mol. Biol. 59, 309–322. doi: 10.1007/s11103-005-8882-0, PMID: 16247559

[ref71] Schwarz E. N. Ruhlman T. A. Sabir J. Hajrah N. Alharbi N. S.. (2015). Plastid genome sequences of legumes reveal parallel inversions and multiple losses of *rps16* in papilionoids. J. Syst. Evol. 53, 458–468. doi: 10.1111/jse.12179

[ref72] Shepherd L. Heenan P. (2017). Evidence for both long–distance dispersal and isolation in the southern oceans: molecular phylogeny of *Sophora sect Edwardsia* (Fabaceae). N. Z. J. Bot. 55, 334–346. doi: 10.1080/0028825X.2017.1353527

[ref73] Shepherd L. Heenan P. (2021). Phylogenomic analyses reveal a history of hybridisation and introgression between *Sophora sect Edwardsia* (Fabaceae) species in New Zealand. N. Z. J. Bot. 1–21. doi: 10.1080/0028825X.2021.1960567 [Epub ahead of print]

[ref74] Sinn B. Sedmak D. D. Kelly L. M. Freudenstein J. (2018). Total duplication of the small single copy region in the angiosperm plastome: rearrangement and inverted repeat instability in *Asarum*. Am. J. Bot. 105, 71–84. doi: 10.1002/ajb2.1001, PMID: 29532923

[ref75] Sun Y. X. Moore M. Meng A. P. Soltis P. Soltis D. Li J.. (2013). Complete plastid genome sequencing of Trochodendraceae reveals a significant expansion of the inverted repeat and suggests a paleogene divergence between the two extant species. PLoS One 8:e60429. doi: 10.1371/journal.pone.0060429, PMID: 23577110 PMC3618518

[ref76] Sun Y. Moore M. Zhang S. Soltis P. S. Soltis D. Zhao T.. (2016). Phylogenomic and structural analyses of 18 complete plastomes across nearly all families of early-diverging eudicots, including an angiosperm-wide analysis of IR gene content evolution. Mol. Phylogenet. Evol. 96, 93–101. doi: 10.1016/j.ympev.2015.12.006, PMID: 26724406

[ref77] Talavera G. Castresana J. (2007). Improvement of phylogenies after removing divergent and ambiguously aligned blocks from protein sequence alignments. Syst. Biol. 56, 564–577. doi: 10.1080/10635150701472164, PMID: 17654362

[ref78] Tonti-Filippini J. Nevill P. Dixon K. Small I. (2017). What can we do with 1000 plastid genomes? The Plant journal: for Cell and Molecular Biology 90, 808–818. doi: 10.1111/tpj.13491, PMID: 28112435

[ref79] Tsoong P. C. Ma C. Y. (1981). A study on the genus *Sophora* Linn. Acta Phytotaxonomica Sinica 19, 1–22.

[ref80] Uribe-Convers, S., Carlsen M. M. Lagomarsino L. P. Muchhala N. (2017). Phylogenetic relationships of *Burmeistera* (Campanulaceae: Lobelioideae): combining whole plastome with targeted loci data in a recent radiation. Mol. Phylogenet. Evol. 107, 551–563. doi: 10.1016/j.ympev.2016.12.011 28011338

[ref81] Vieira L. N. Faoro H. Rogalski M. Fraga H. P., Cardoso, R. L. de Souza E. M.., (2014). The complete chloroplast genome sequence of *Podocarpus lambertii*: genome structure, evolutionary aspects, gene content and SSR detection. PLoS One 9, e90618. doi: 10.1371/journal.pone.0090618, PMID: 24594889 PMC3942463

[ref82] Wang Y. H. Qu X. J. Chen S. Y. Li D. Yi T. S. (2017). Plastomes of Mimosoideae: structural and size variation, sequence divergence, and phylogenetic implication. Tree Genetics Genomes 13, 1–18. doi: 10.1007/s11295-017-1124-1

[ref83] Wang Y. H. Wicke S. Wang H. Jin J. J. Chen S. Y. Zhang S. D.. (2018). Plastid genome evolution in the early–diverging legume subfamily Cercidoideae (Fabaceae). Front. Plant Sci. 9, 138. doi: 10.3389/fpls.2018.00138, PMID: 29479365 PMC5812350

[ref84] Wei F. Tang D. F. Wei K. H. Qin F. Li L. X. Lin Y.. (2020). The complete chloroplast genome sequence of the medicinal plant *Sophora tonkinensis*. Sci. Rep. 10, 12473. doi: 10.1038/s41598-020-69549-z, PMID: 32719421 PMC7385175

[ref85] Wick R. Schultz M. Zobel J. Holt K. (2015). Bandage: interactive visualization of de novo genome assemblies. Bioinformatics 31, 3350–3352. doi: 10.1093/bioinformatics/btv383, PMID: 26099265 PMC4595904

[ref86] Wicke S. Schneeweiss G. Depamphilis C. Müller K. Quandt D. (2011). The evolution of the plastid chromosome in land plants: gene content, gene order, gene function. Plant Mol. Biol. 76, 273–297. doi: 10.1007/s11103-011-9762-4, PMID: 21424877 PMC3104136

[ref87] Win P. P. Li X. Chen L. Tan Y. H. Yu W. B. (2020). Complete plastid genome of two *Dalbergia* species (Fabaceae), and their significance in conservation and phylogeny. Mitochondrial DNA Part B 5, 1967–1969. doi: 10.1111/jse.12598

[ref88] Wojciechowski M. F. W. Sanderson M. J. Steel K. P. Liston A. (2000). “Molecular phylogeny of the “temperate herbaceous tribes” of papilionoid legumes: a supertree approach,” in Advances in Legume Systematics 9. eds. Herendeen P. Bruneau A. (Kew: Royal Botanic Garden), 277–298.

[ref89] Xu J. Feng D. Song G. Wei X. Chen L. Wu X.. (2008). The first intron of rice EPSP synthase enhances expression of foreign gene. Sci. China Ser. C Life Sci. 46, 561–569. doi: 10.1360/02yc0120, PMID: 18758713

[ref90] Xu X. Wang D. (2020). Comparative chloroplast genomics of *Corydalis* species (Papaveraceae): evolutionary perspectives on their unusual large scale rearrangements. Front. Plant Sci. 11:600354. doi: 10.3389/fpls.2020.600354, PMID: 33584746 PMC7873532

[ref91] Zha X. Wang X. Li J. Gao F. Zhou Y. (2020). Complete chloroplast genome of *Sophora alopecuroides* (Papilionoideae): molecular structures, comparative genome analysis and phylogenetic analysis. J. Genet. 99, 13. doi: 10.1007/s12041-019-1173-3 32089532

[ref92] Zhang L. Zheng Y. Deng H. Z. Liang L. Peng J. (2014). Aloperine induces G2/M phase cell cycle arrest and apoptosis in HCT116 human colon cancer cells. Int. J. Mol. Med. 33, 1613–1620. doi: 10.3892/ijmm.2014.1718, PMID: 24682388

[ref93] Zhang R. Wang Y. H. Jin J. Stull G. W. Bruneau A. Cardoso D.. (2020a). Exploration of plastid phylogenomic conflict yields new insights into the deep relationships of Leguminosae. Syst. Biol. 69, 613–622. doi: 10.1093/sysbio/syaa013, PMID: 32065640 PMC7302050

[ref94] Zhang Y. An D. Li C. Zhao Z. Wang W. (2020b). The complete chloroplast genome of greater duckweed (*Spirodela polyrhiza* 7498) using PacBio long reads: insights into the chloroplast evolution and transcription regulation. BMC Genomics 21, 76. doi: 10.1186/s12864-020-6499-y, PMID: 31992185 PMC6986005

[ref95] Zhao K. Li L. Quan H. Yang J. B. Zhang Z. Liao Z.. (2020). Comparative analyses of chloroplast genomes from 14 *Zanthoxylum* species: identification of variable DNA markers and phylogenetic relationships within the genus. Front. Plant Sci. 11:605793. doi: 10.3389/fpls.2020.605793, PMID: 33519856 PMC7838127

[ref96] Zhu A. Guo W. Gupta S. Fan W. Mower J. P. (2016). Evolutionary dynamics of the plastid inverted repeat: the effects of expansion, contraction, and loss on substitution rates. New Phytol. 209, 1747–1756. doi: 10.1111/nph.13743, PMID: 26574731

