# Supplementary material for: Comparative Chloroplast Genomics of Sophora Species: Evolution and Phylogenetic Relationships in the Early-Diverging Legume Subfamily Papilionoideae (Fabaceae)
Source: Front Plant Sci. 2021 Dec 16;12:778933. doi: 10.3389/fpls.2021.778933 (PMC8716937; doi:10.3389/fpls.2021.778933)
Supplement: Supplementary file 1 [file Data_Sheet_1.docx]

Supplementary Material

# Supplementary Figure

Figure S1. (A) Nucleotide content of the IR, LSC, and SSC regions of 14 *Sophora* species; (B) The GC (%) composition in different positions (Total, IR, LSC, and SSC) of the plastid genomes of 14 *Sophora* species


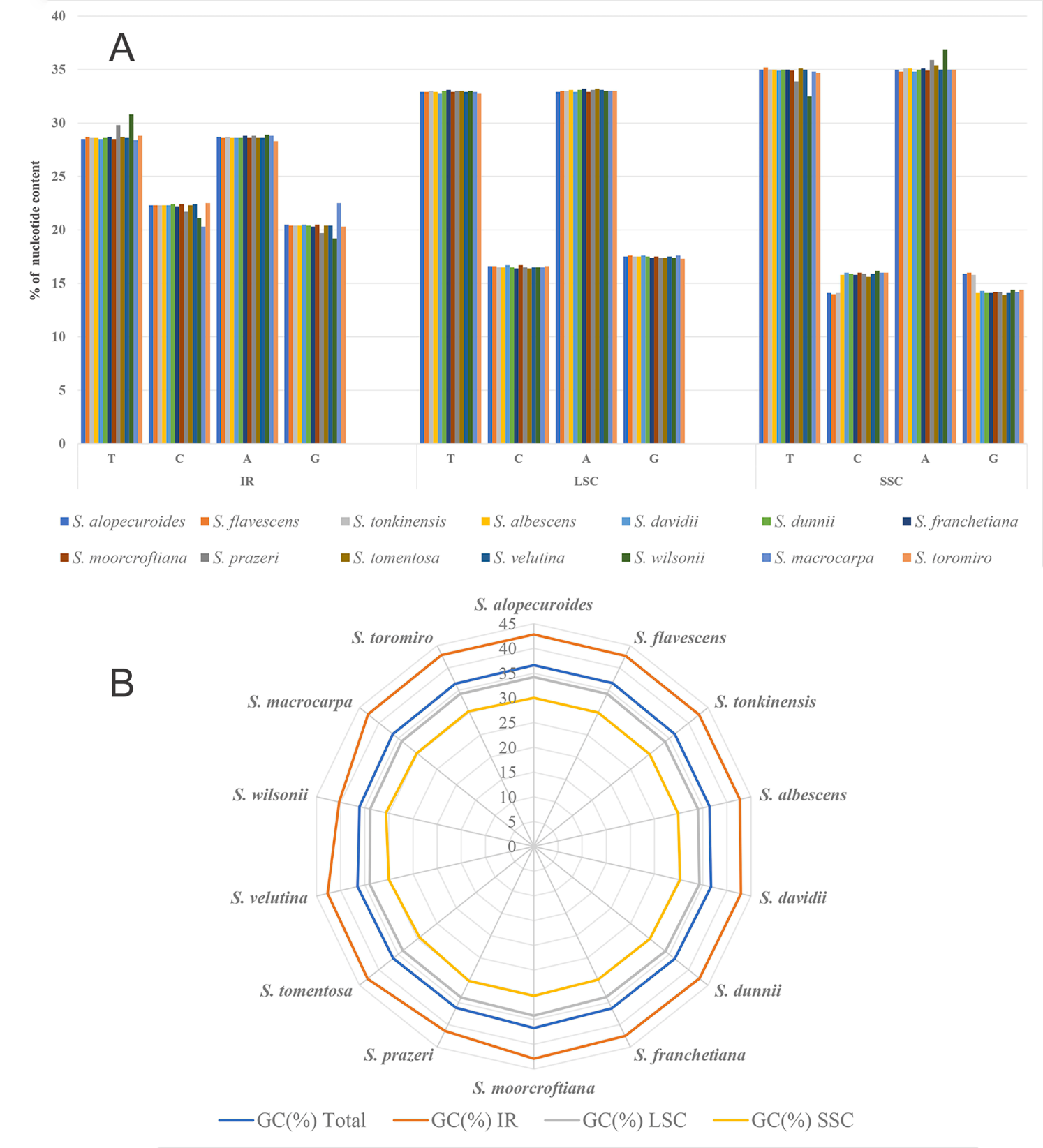


Figure S2. Phylogenetic tree obtained using the Maximum Likelihood (ML) method of the plastid genomes of 39 taxa. The full support values are not indicated


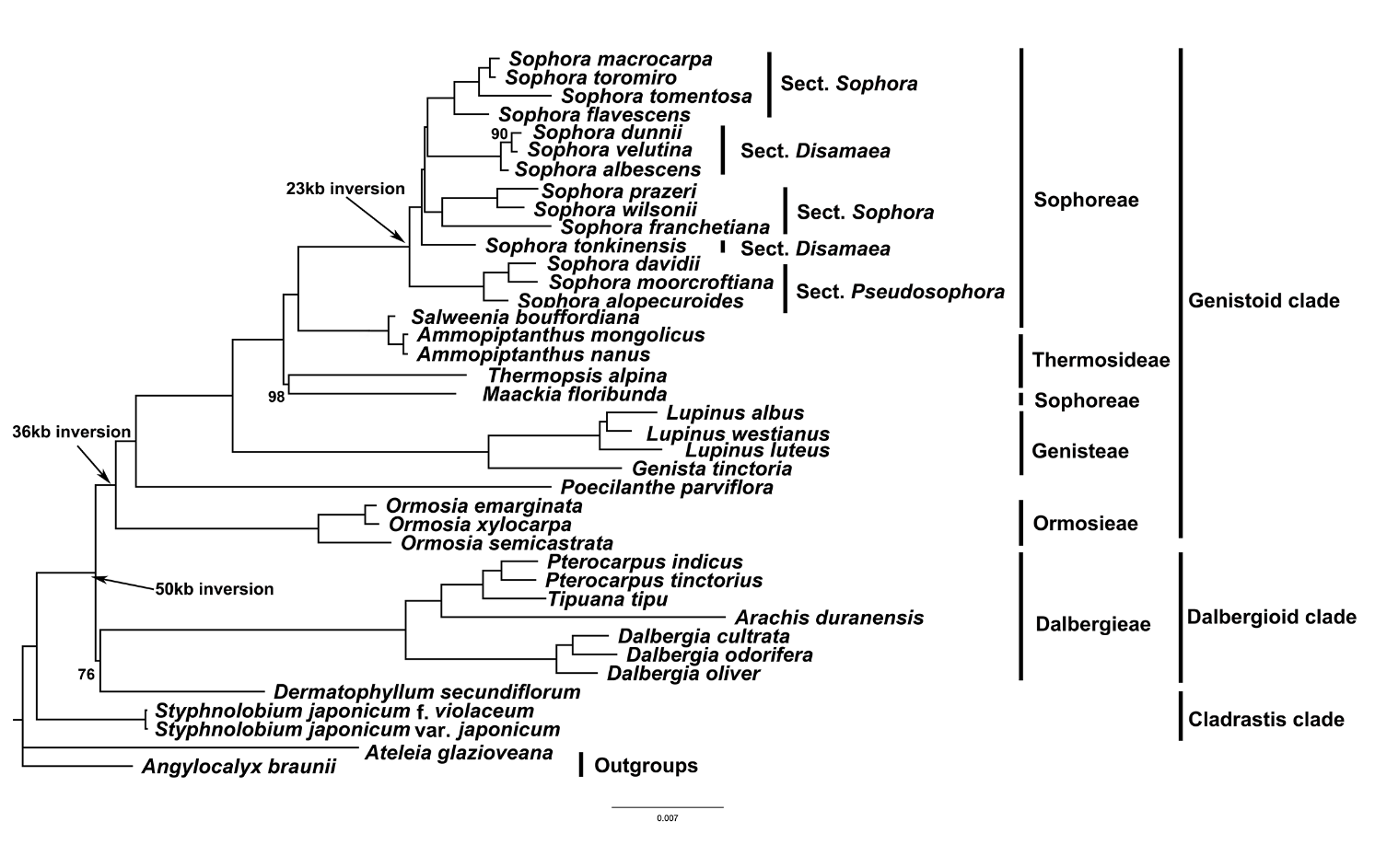


Figure S3. Phylogenetic tree obtained using the Maximum Likelihood (ML) method of the CDS of 39 taxa. The full support values are not indicated


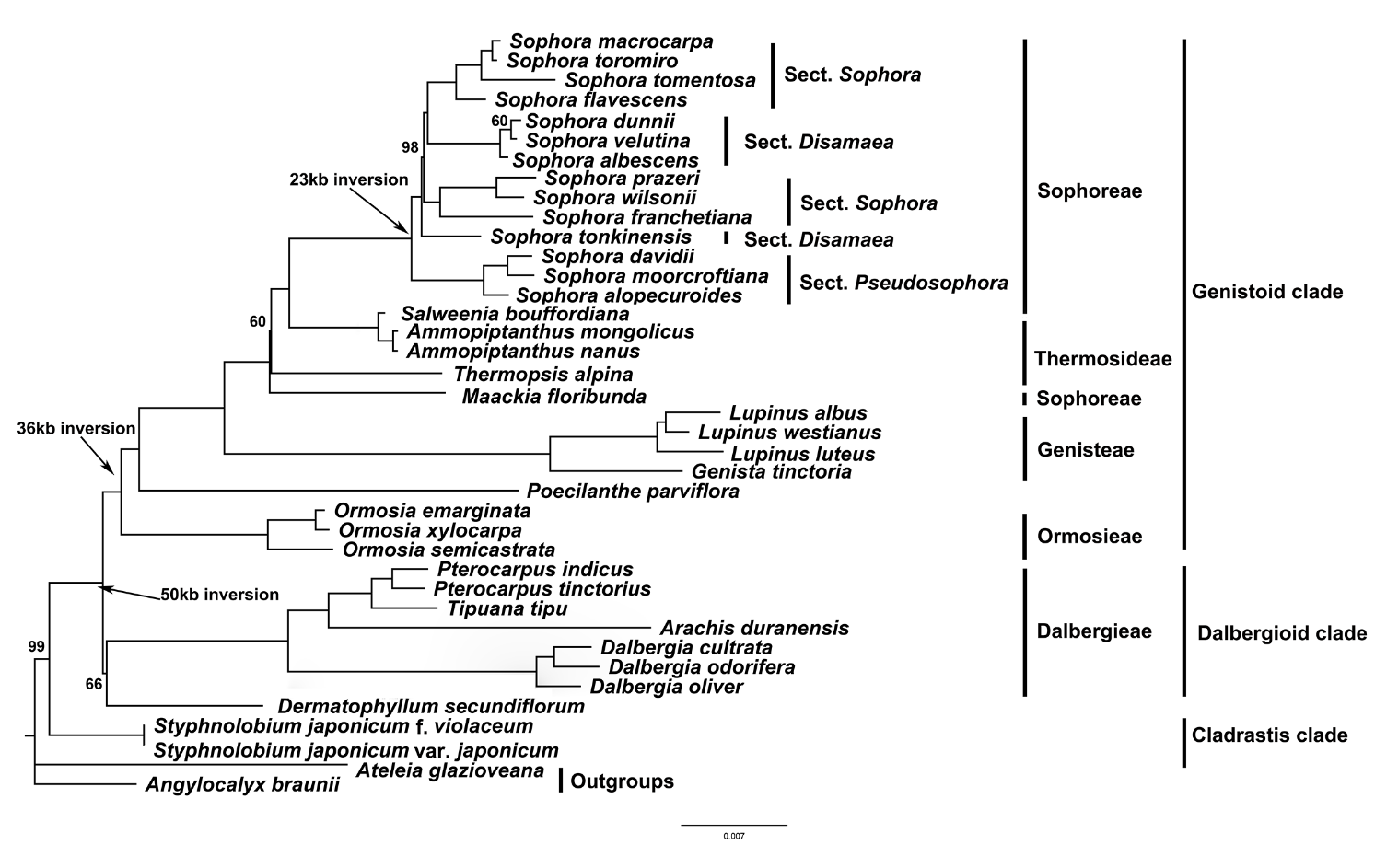


Figure S4. Phylogenetic tree obtained using the Bayesian inference (BI) method of the CDS of 39 taxa. The full support values are not indicated


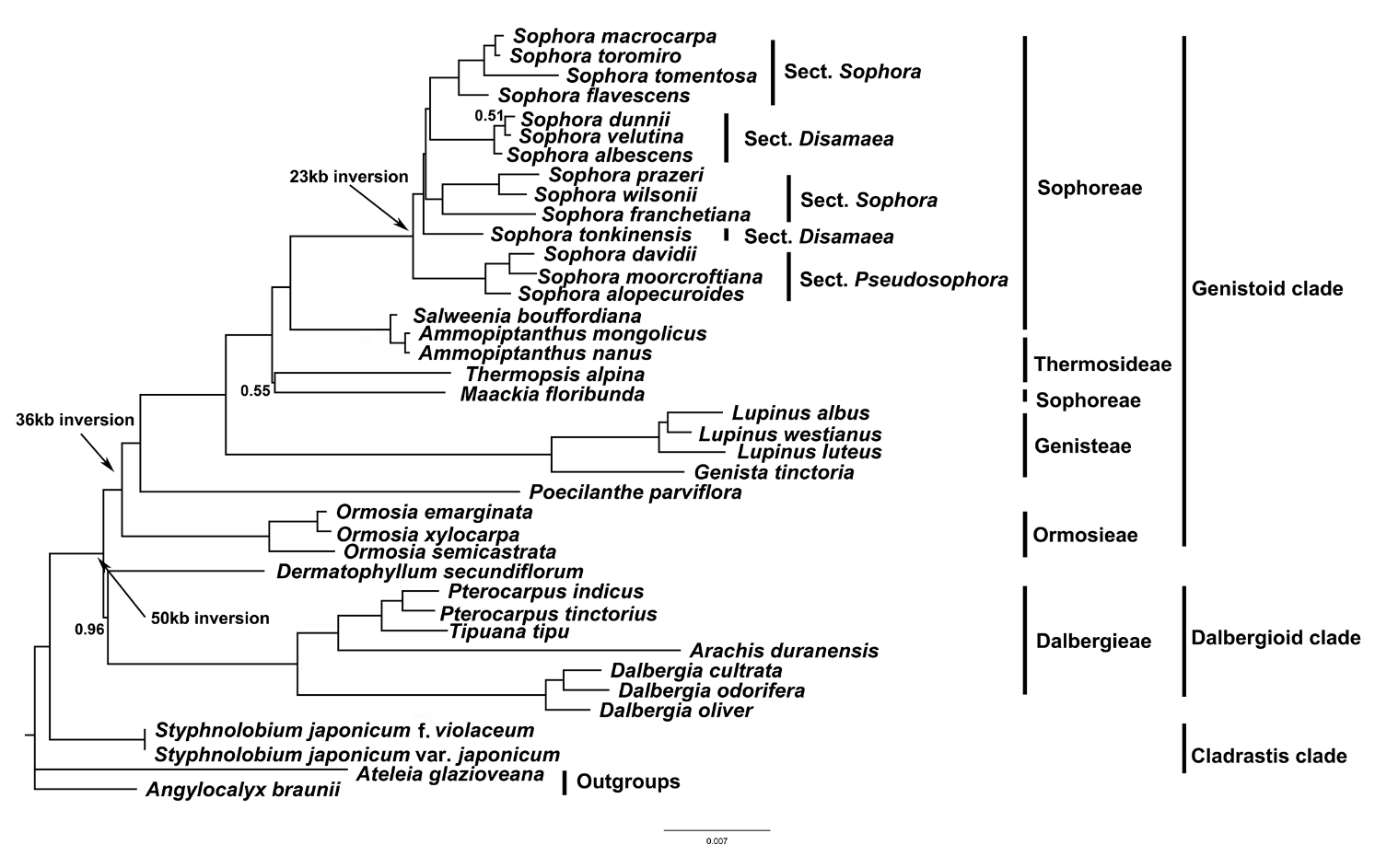


Figure S5. Phylogenetic tree obtained using the Maximum Likelihood (ML) and Bayesian inference (BI) methods of the seven suitable polymorphism loci of 39 taxa. The full support values are not indicated


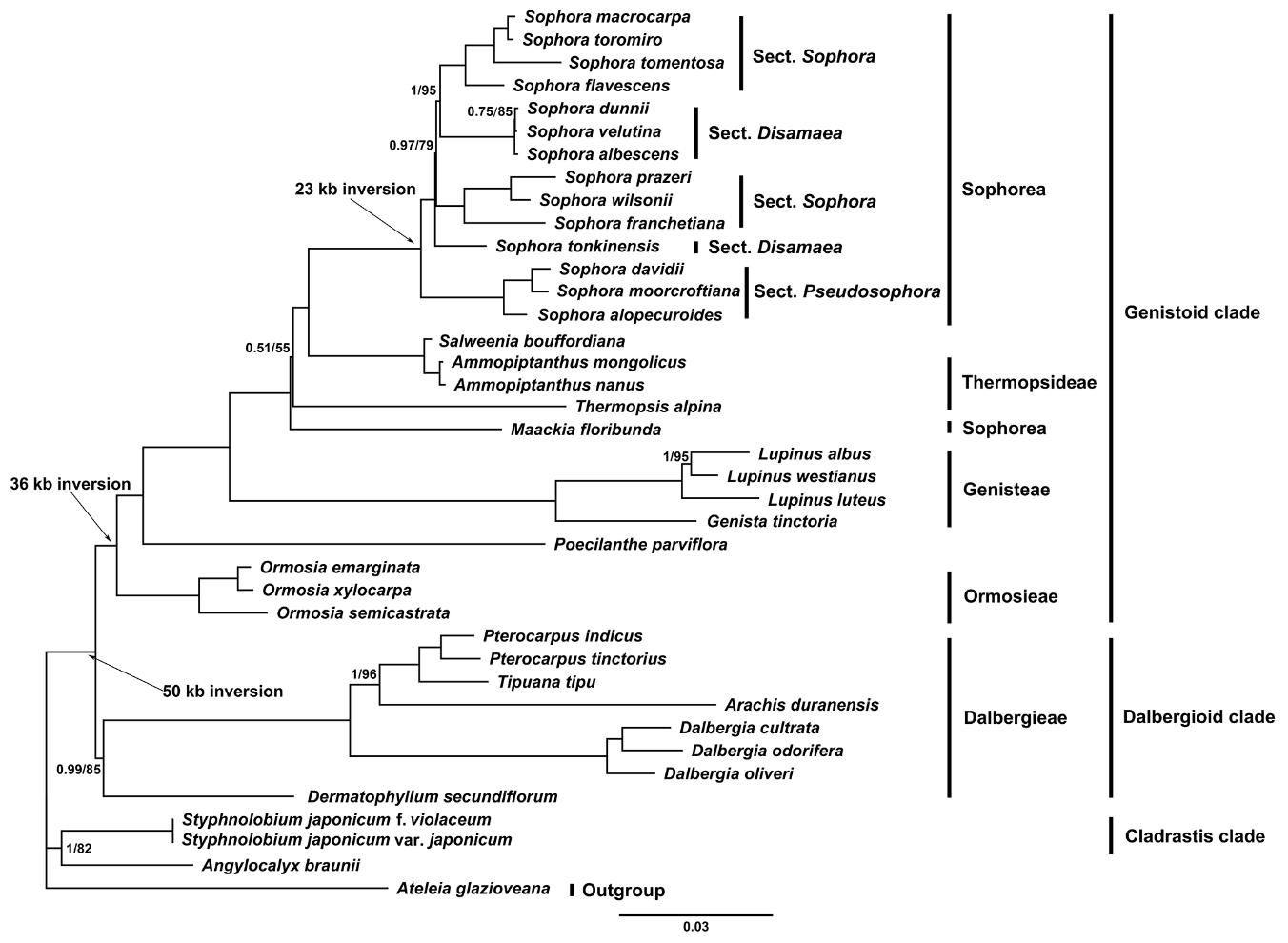


# Supplementary Table

**Table S1**. List of species used for phylogenetic tree construction

**Table S2**. Summary of the characteristics of *Sophora* chloroplast genomes

**Table S3**. Nucleotide content of IR, LSC, and SSC in the chloroplast genomes of fourteen *Sophora* species

**Table S4.** List of annotated genes in *Sophora* chloroplast genomes

**Table S5**. List of intron numbers of genes in the chloroplast genomes of *Sophora*

**Table S6**. SSR comparison of fourteen *Sophora* species

**Table S7**. Repeat sequence analysis of fourteen *Sophora* chloroplast genomes with different Hamming distances

**Table S8**. Number of repeats in the IR, LSC, and SSC of fourteen *Sophora* species

**Table S9.** Number of nucleotides in variable site and nucleotide diversity analysis among 14 *Sophora* species

**Table S10.** Thirty regions of highly variable sequences in 14 *Sophora* species

**Table S11.** LSRs in Sophora species

# Supplementary Table Reference

Choi, I. S., and Choi, B. (2017). The distinct plastid genome structure of *Maackia fauriei* (Fabaceae: Papilionoideae) and its systematic implications for genistoids and tribe Tr. Sophoreae. *PLoS One* 12, e0173766. doi: [10.1371/journal.pone.0173766](file:///C:\Users\xubo\Desktop\2021年4月近期待投稿论文\Liao%20et%20al.,%20Sophora%20cp%20genome\10.1371\journal.pone.0173766)

Duan, N., Deng, Y., Liu, Y., Zhang, Y., Zhang, L. G., Wang, C. Y. et al., (2019). The complete chloroplast genome of *Sophora alopecuroides* (Fabaceae). *Mitochondrial DNA Part B* 4(1):1336–1337. doi: <10.1080/23802359.2019.1596760>

Liu, H., Su, Z., Yu, S., Liu, J., Yin, X., Zhang, G., et al. (2019a). Genome comparison reveals mutation hotspots in the chloroplast genome and phylogenetic relationships of *Ormosia* species. *BioMed Research International*, 1–11. doi: <10.1155/2019/7265030>

Lu, Y. Z., Li, W. Q, Xie, X. M., Zheng, Y. Q., and Li, B. (2018). The complete chloroplast genome sequence of *Sophora japonica* var. *violacea*: gene organization and genomic resources. *Conservation Genetics Resources* 10, 1–4. doi:<10.1007/s12686-017-0748-7>

Martin, G., Rousseau–Gueutin, M., Cordonnier, S., Lima, O., Michon–Coudouel, S., Naquin, D., et al. (2014). The first complete chloroplast genome of the Genistoid legume *Lupinus luteus*: evidence for a novel major lineage–specific rearrangement and new insights regarding plastome evolution in the legume family. *Annals of Botany* 113**,** 1197–1210. doi: [10.1093/aob/mcu050](file:///C:\Users\xubo\Desktop\2021年4月近期待投稿论文\Liao%20et%20al.,%20Sophora%20cp%20genome\10.1093\aob\mcu050)

Ni, X. L., Li, B. F., Jia, G. L., Song, N. P., and Li, Z. G. (2019). Characterization of the complete chloroplast genome of *Ammopiptanthus mongolicus* (Papilionoideae), a rare and Endangered plant to China. *Mitochondrial DNA Part B* 4,1886-1887. doi: <10.1080/23802359.2019.1613186>

Pezoa, I., Villacreses, J., Rubilar, M., Pizarro, C., Galleguillos, M. J., Ejimentewica, T. et al., (2021). Generation of chloroplast molecular markers to differentiate *Sophora toromiro* and its hybrids as a first approach to its reintroduction in Rapa Nui (Easter Island). *Plants* 10(2), 342. doi: <10.3390/plants10020342>

Wariss, H. M., Yi, T. S., Wang, H., and Zhang, R. (2018). The chloroplast genome of a rare and an endangered species *Salweenia bouffordiana* (Leguminosae) in China. *Conservation Genetics Resources* 10, 405–407. doi: <10.1007/s12686-017-0836-8>

Win, P. P., Li, X., Chen, L., Tan, Y. H., and Yu, W. B. (2020). Complete plastid genome of two *Dalbergia* species (Fabaceae), and their significance in conservation and phylogeny. *Mitochondrial DNA Part B* 5**,** 1967–1969. doi: [10.1111/jse.12598](file:///C:\Users\xubo\Desktop\2021年4月近期待投稿论文\Liao%20et%20al.,%20Sophora%20cp%20genome\10.1111\jse.12598)

Zhang, R., Wang, Y. H., Jin, J., Stull, G. W., Bruneau, A., Cardoso, D., et al. (2020a). Exploration of plastid phylogenomic conflict yields new insights into the deep relationships of Leguminosae. *Systematic Biology* 69, 613–622. doi: <10.1093/sysbio/syaa013>

Zhang, W. L., Li, L., and Li, G. H. (2018). Characterization of the complete chloroplast genome of shrubby *Sophora* (*Sophora flavescens* Ait.). *Mitochondrial DNA Part B* 3(2):1282-1283. doi: <10.1080/23802359.2018.1532839>
